# Silicon Carbide Microstrip Radiation Detectors

**DOI:** 10.3390/mi10120835

**Published:** 2019-11-30

**Authors:** Donatella Puglisi, Giuseppe Bertuccio

**Affiliations:** 1 Department of Electronics, Information and Bioengineering, Politecnico di Milano, Campus Como, 22100 Como, Italy; giuseppe.bertuccio@polimi.it; 2 Department of Physics, Chemistry and Biology, Sensor and Actuator Systems, Linköping University, 58183 Linköping, Sweden; 3 Italian National Institute of Nuclear Physics (INFN), Section Milano, 20133 Milan, Italy

**Keywords:** silicon carbide, semiconductor radiation detector, microstrip detector

## Abstract

Compared with the most commonly used silicon and germanium, which need to work at cryogenic or low temperatures to decrease their noise levels, wide-bandgap compound semiconductors such as silicon carbide allow the operation of radiation detectors at room temperature, with high performance, and without the use of any bulky and expensive cooling equipment. In this work, we investigated the electrical and spectroscopic performance of an innovative position-sensitive semiconductor radiation detector in epitaxial 4H-SiC. The full depletion of the epitaxial layer (124 µm, 5.2 × 10^13^ cm^−3^) was reached by biasing the detector up to 600 V. For comparison, two different microstrip detectors were fully characterized from −20 °C to +107 °C. The obtained results show that our prototype detector is suitable for high resolution X-ray spectroscopy with imaging capability in a wide range of operating temperatures.

## 1. Introduction

The concept of using compound semiconductors as radiation detectors was introduced in 1945 by Van Heerden [1,2], who was the first to be able to detect alpha and gamma rays with solid-state radiation counters. His pioneering results gave rise to a new class of radiation detectors, which is now commonly known as semiconductor detectors. Compared to gas detectors, semiconductor detectors require much lower average energies for the creation of electron-hole pairs (30 eV for gas [3], 3.7 eV for Si [4], 7.8 eV for 4H-SiC [5]), which bring higher energy resolution in radiation spectroscopy [6,7]. Since the 1960s, the most commonly used semiconductor materials have been high-purity silicon (Si) and germanium (Ge), the main limitation of which is that they must operate at liquid nitrogen temperature. Since the 1990s, intense research activity has been carried out on other semiconductors for manufacturing detectors able to operate at room temperature, such as gallium arsenide (GaAs), cadmium telluride (CdTe), and cadmium zinc telluride (CdZnTe) [8,9,10,11,12]. In the last two decades, silicon carbide (SiC) has obtained increasing interest in the field of radiation detectors due to the achievement of a high purity level in the crystal structure and considerable thickness (>100 μm) in the epitaxial layer. This finally achieved recognition for semiconductor detectors as a real alternative to Si-based radiation detectors, which present possibilities but also limitations at and above room temperature, as well as in high-radiation environments [13,14]. There are certain properties that make SiC especially suitable for the realization of ionizing radiation detectors. Thanks to the wide energy bandgap of the polytype 4H-SiC (3.26 eV), which is three times higher than that of Si (1.12 eV), electronic devices fabricated in such material can operate at extremely high temperatures without suffering from negative effects, due to thermally generated charge carriers [15]. Silicon carbide radiation detectors benefit from this property because the wide energy bandgap allows the achievement of very low leakage currents, i.e., very low noise levels, even at the high electric fields applied during their operation. Moreover, the high thermal conductivity of 4H-SiC (3.8 W/cm°C) enables SiC devices to dissipate large amounts of excess generated heat, which would cause a temperature increase, responsible for degradation of the device’s performance. High thermal conductivity is useful for increasing the radiation hardness of the detector, as well as for controlling the operating temperature when the front-end electronics are close to, or in contact with, the detector [16]. Furthermore, SiC can withstand an internal electric field over eight to ten times greater than GaAs or Si (2 MV/cm for 4H-SiC vs. 0.4 MV/cm for GaAs or 0.3 MV/cm for Si) without undergoing avalanche breakdown. This property enables the fabrication of very high-voltage devices [17]. In the case of X-ray detection and spectroscopy, the high breakdown field of 4H-SiC allows, in principle, the detector to work always in the regime of saturated-electron and hole-drift velocities, independently of the detector’s active region width. When this operation condition is coupled with epitaxial material of high crystalline quality, a full and fast charge collection can be expected [16], as well as a high sensitivity, as already demonstrated [18]. Such properties allow SiC-based devices to be operated without any costly, bulky, and power-consuming cooling systems, as in the case of Si- or Ge-based devices, while maintaining an excellent signal-to-noise ratio over a wide range of temperatures. This leads to notable advantages in terms of the lower cost, more compact size, lighter weight, lower power consumption, and higher performance of SiC detectors. Further explanation of the electrical properties of SiC in connection with the ionizing detector performance benefits can be found in [16].

Microstrip detectors find application where the position of the radiation interaction is necessary information for the physical process to be studied. The advantage of using microstrips with respect to other position-sensitive detectors, such as pixel detectors, is a lower number of readout channels. Several microstrip detectors have been developed in Si for high-energy physics, or in Ge, CdTe and GaAs for X-ray spectroscopy [19,20,21,22]. In this work, we investigated the electrical and spectroscopic performance of two innovative position-sensitive radiation detectors in epitaxial 4H-SiC, using microstrip geometry. The detectors were characterized in detail at different temperatures and applied bias voltages. The obtained results are presented and discussed in the following sections.

## 2. Materials and Methods

**Figure 1:**
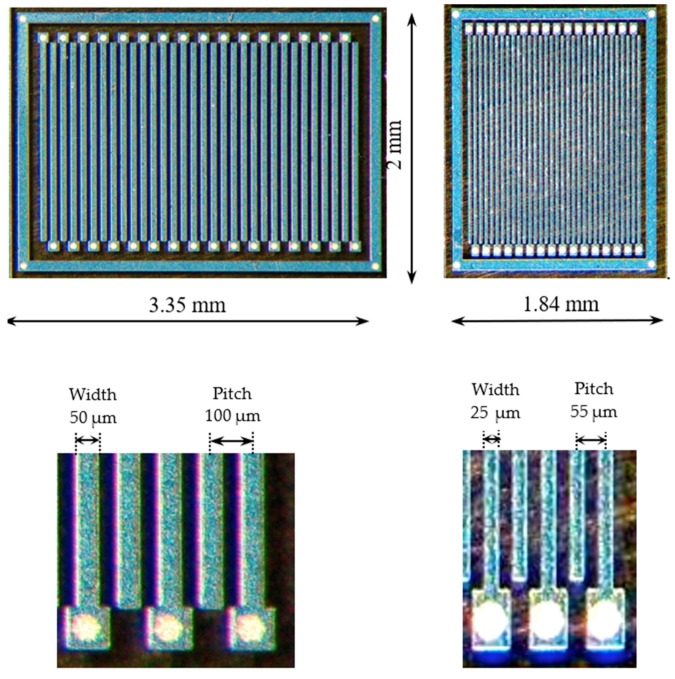
Photographs of the two microstrip detectors used in this work, together with a detail of their peripheral regions with bonding pads.

**Figure 2:**
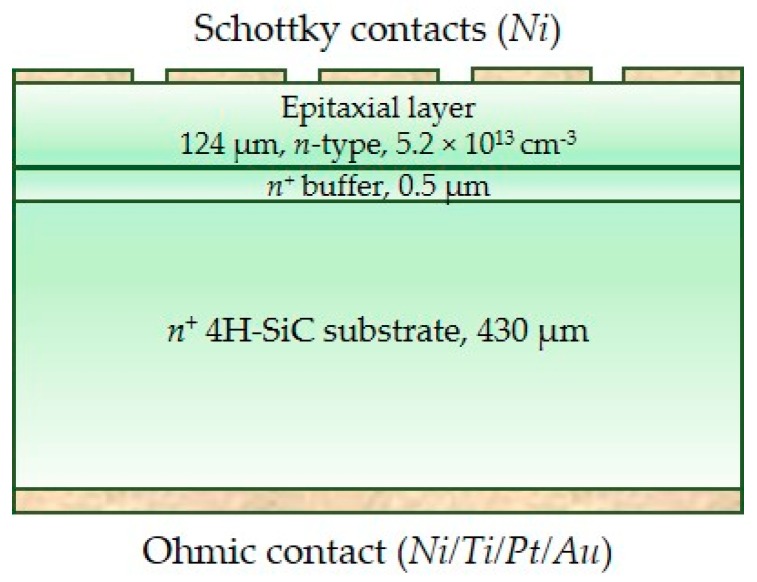
Cross-sectional view of the 4H-SiC microstrip structure.

**Figure 3:**
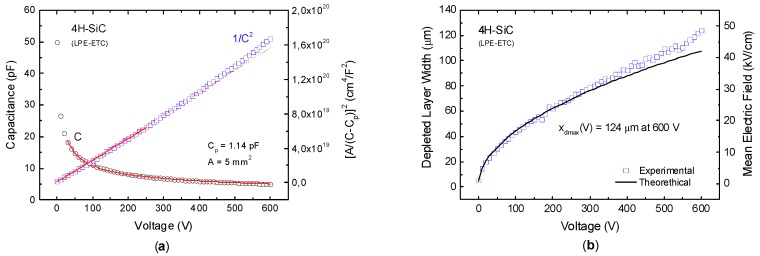
(**a**) Capacitance–voltage (C–V) and 1/C^2^–V per unit area characteristics. From the fit curve, the mean value of the donor concentration is expected to be (5.56 ± 0.05) × 10^13^ cm^−3^; (**b**) depleted layer and mean electric field as a function of applied voltage, as derived from C–V measurements. The theoretical result is obtained using 5.56 × 10^13^ cm^−3^ as the mean value of the doping concentration.

Two different designs of silicon carbide microstrip detectors have been realized on top of two-inch high-purity epitaxial 4H-SiC wafer produced by LPE Epitaxial Technology Center [23]. Each detector consists of 32 strips with a length of 2 mm, a width of either 25 μm (SM1) or 50 μm (SM3), and a pitch of either 55 μm (SM1) or 100 μm (SM3)—see Figure 1. Each of these strips can be read out independently by a front-end electronics channel, and therefore behaves as a separate detector. A cross-sectional view of the 4H-SiC microstrip structure is shown in Figure 2. The SiC epitaxial layer, which is the active region of the detector, has a maximum thickness of 124 µm, as experimentally measured (Figure 3).

The application of a reverse bias at the common back electrode (ohmic contact) creates a depletion region, *x_d_*, depending on the applied bias, *V_R_*, and the residual doping (donor) concentration, N_D_, within the material, according to

(1) $$x_{d}=\sqrt{\frac{2\varepsilon_{0}\varepsilon_{r}}{qN_{D}}\left(\psi_{bi}-V_{R}-\frac{kT}{q}\right)}$$

where $\psi_{bi}$ is the built-in potential and the term $\frac{kT}{q}$ arises from the contribution of the majority-carrier distribution tail [24].

The residual doping concentration depends on the homogeneity of the epitaxial layer. The donor concentration profile, *N_D_*(*x*), was determined as a function of the depleted layer width from capacitance-voltage measurements, as described in Section 3.

## 3. Results

### 3.1. Electrical Characterization

#### 3.1.1. Capacitance–Voltage Characterization

**Figure 4:**
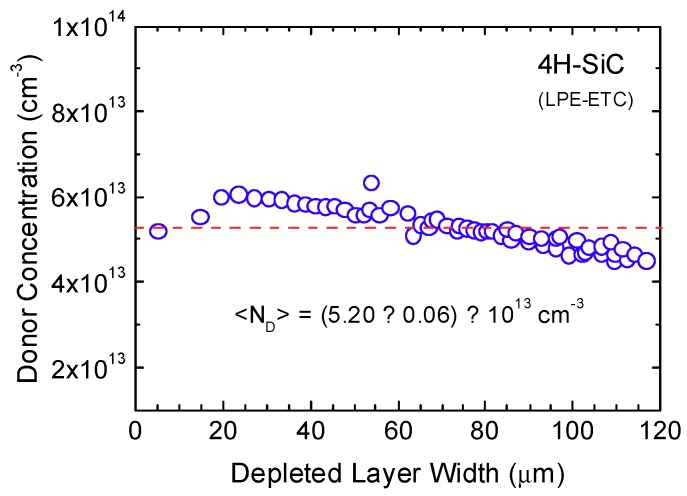
Donor concentration profile as a function of the depleted layer width. The full depletion of 124 μm was reached at 600 V. A mean value of <N_D_> = 5.2 × 10^13^ cm^−3^ was determined.

Capacitance–voltage (C–V) measurements were carried out up to 600 V at 25 °C, in order to determine the donor-concentration profile of the epitaxial layer (Figure 3a). The detector was placed in a test fixture Agilent 16065A connected to an Agilent 4284A Precision LCR Meter (Santa Clara, CA, USA). A Keithley 2410 voltage source (Cleveland, OH, USA), operating in the four-wire connection mode, was used to bias the device and measure the applied voltage. The measurement was performed with a 100 mV AC signal at 100 kHz. The donor-concentration profile as a function of the depleted layer width was determined from the slope of a 1/C^2^–V curve, according to [24], see Figure 3a. Please note that the C–V measurements were performed using a 4H-SiC Schottky diode with area *A* = 5 mm^2^, produced from the same wafer. A full depletion of 124 μm was reached, polarizing the detector up to 600 V (Figure 3b). A mean value of <N_D_> = (5.20 ± 0.06) × 10^13^ cm^−3^ was determined (Figure 4).

#### 3.1.2. Statistical Leakage–Current Distribution

**Figure 5:**
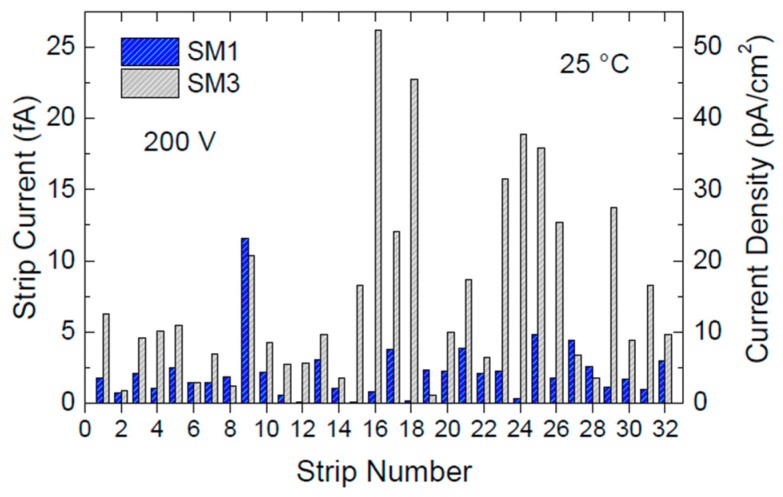
Current and current density measured at 25 °C and 200 V on the 32 strips of the two microstrip detectors, SM1 and SM3. Current values of few fA (current densities of low pA/cm^2^) were measured on all strips.

Current–voltage (I–V) measurements were carried out on each of the 32 strips of two different SiC detectors, SM1 and SM3, at room temperature (Figure 5). The two detectors were biased at 100 V and 200 V from the back ohmic contact using a Keithley 2410 source meter, whereas the current of each strip was measured connecting a Keithley 6430 electrometer to the front, rectifying the Schottky contact. The guard electrode surrounding the microstrips was kept to ground to collect the parasitic current generated at the device’s chip edges. Figure 5 shows the current and current density values for each of the 32 strips of the two different microstrip detectors as measured at 25 °C and 200 V. Such bias voltage generates an inner electric field of about 30 kV/cm. Ultra-low current mean values of 2.2 fA and 7.6 fA were measured for the two microstrips under test, corresponding to current densities of 4.4 pA/cm^2^ and 15.2 pA/cm^2^.

#### 3.1.3. Temperature Dependence

**Figure 6:**
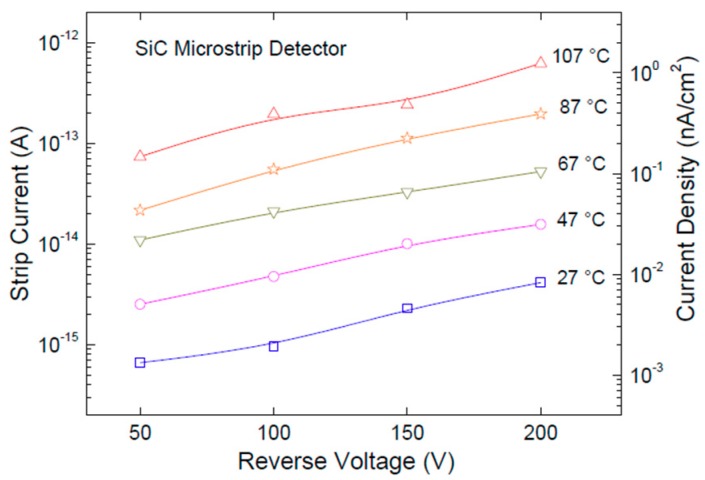
Current and current density dependence from temperature in the range 27 to 107 °C.

The temperature dependence of the strip current as a function of reverse-bias voltage is shown in Figure 6. From now on, only results obtained with the detector SM1 are presented. In order to perform this measurement, the device was attached to a Teflon circuit board using a silver conductive glue. Electrical contacts were established using 25 μm gold wire-bonding connections. Measurements were acquired, biasing up to 200 V the back contact with the Keithley 2410 source meter and reading the currents from the Keithley 6430 electrometer. Tests were carried out inside an environmental chamber, setting the temperature at 27 °C, 47 °C, 67 °C, 87 °C, and 107 °C, and monitoring it by means of a thermocouple placed near to the device. During each measurement, the temperature changes were monitored within ±0.1 °C. The current density was calculated considering a strip active area of 5 × 10^−4^ cm^−2^.

Such a thermally activated process is described by the Arrhenius plot that, according to the emission theory, is expressed by

(2) $$I=I_{0}\cdot \exp\left[\left(-\frac{E_{A}}{kT}\right)\cdot \left(1-\frac{T}{T_{0}}\right)\right]$$

where *I*_0_ is the saturation current, *T*_0_ is the room temperature, and *E*_A_ is the activation energy [5,25].

**Figure 7:**
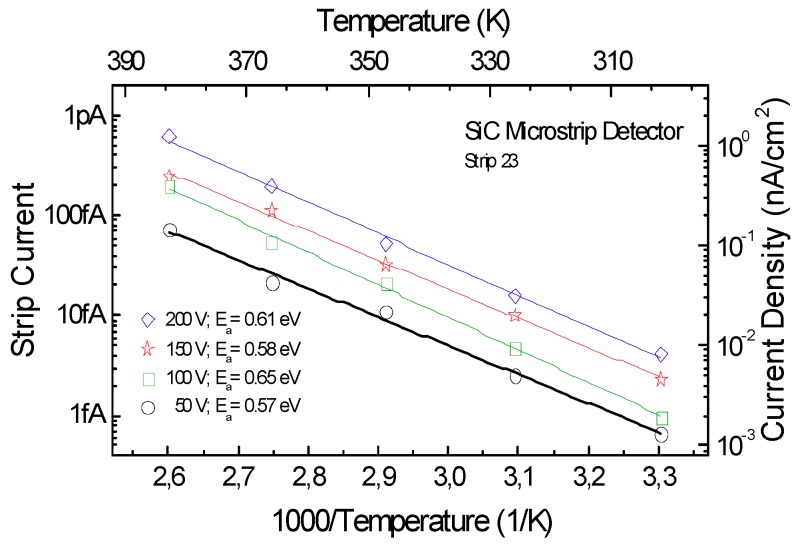
Arrhenius plots of the leakage current (and current density) versus the reverse of temperature at four different voltages. The activation energy values are from 0.57 eV to 0.65 eV in the voltage range 50 to 200 V.

Figure 7 shows the Arrhenius plots of the leakage current as a function of 1000/*T* at four different reverse voltages, i.e., 50 V, 100 V, 150 V, and 200 V. The activation energy is given by the slope of linear fit of data. Values from 0.57 eV to 0.65 eV were calculated in the voltage range 50 V to 200 V. According to the literature, these values refer to major deep levels (*Z*_1/2_ center) within the bandgap [26,27,28].

#### 3.1.4. Interstrip Resistance Measurements

Current–voltage measurements shown in previous sections refer to the characterization of single strips, considering that the measured current arrived only from the back contact of the device, and possible latent currents from adjacent strips were negligible. In order to determine the bias limit condition so that two adjacent strips can be considered isolated, we measured the interstrip resistance. 

**Figure 8:**
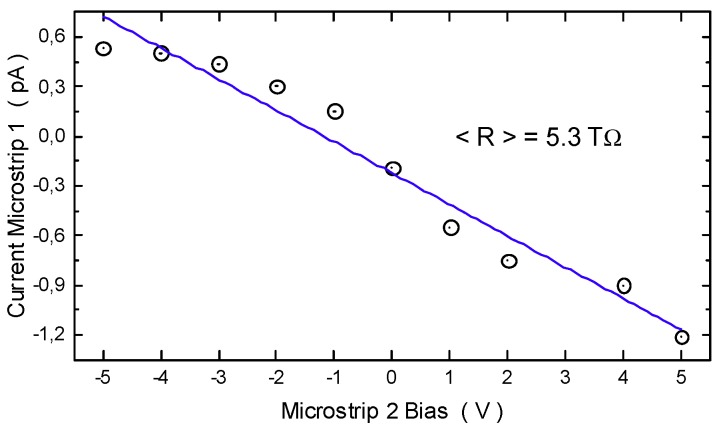
Mean value of resistance between two adjacent strips.

Interstrip resistance measurements were carried out by measuring the current between two consecutive strips, keeping the back contact at 100 V and the guard at 0 V. One of the two strips (microstrip 2 in Figure 8) is biased from −5 V to +5 V while the current of the other strip (microstrip 1 in Figure 8), kept at 0 V, is measured by an electrometer. We repeated the test on three couples of strips. The negative slope shown in Figure 8 is due to the application of the bias voltage to microstrip 2 while measuring the current at microstrip 1. The mean value of resistance between two adjacent strips of SM1 resulted in 5.3 TΩ.

### 3.2. X-Ray Spectroscopy

#### 3.2.1. Room Temperature

**Figure 9:**
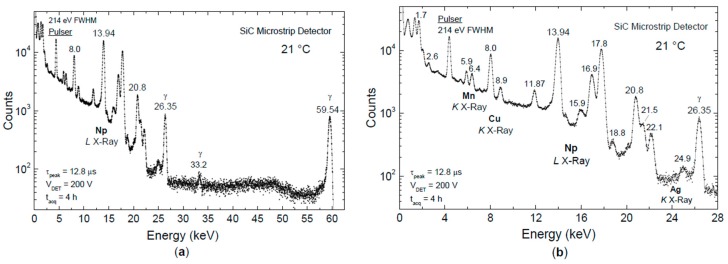
(**a**) X-ray spectrum from a ^241^Am source acquired at 21 °C using the SiC microstrip detector SM1 and an ultra-low noise front-end (PRE5 no. 3); (**b**) detailed X-ray spectroscopy in the energy range 0 to 28 keV.

The detector was irradiated with a 397 kBq ^241^Am source placed at a few cm from the detector surface. Figure 9a shows a ^241^Am spectrum acquired using a SiC microstrip detector at 21 °C. The spectrum was acquired at 200 V reverse-bias condition using 12.8 µs peaking time in the triangular signal processing. The pulser line width is 214 eV full width at half maximum (FWHM) corresponding to an equivalent noise charge of 11.6 electrons root mean square (rms). Figure 9b shows a detail of the same measurement up to 28 keV. Several X-ray lines from Mn, Cu, Np, and Ag can be clearly distinguished with a very good resolution, i.e., enough to separate the *K* and *L* lines of neighboring elements. Conventionally, the energy resolution, that is the FWHM, is specified for the Mn *Kα* peak at 5.9 keV, which is 213 eV for our SiC microstrip detector at room temperature (Figure 9). It is notable that Si(Li) and silicon drift detectors can achieve 130–150 eV FWHM, and Ge detectors can even achieve 115 eV FWHM for the Mn *Kα* peak at 5.9 keV, but with liquid-nitrogen cooling [29].

**Figure 10:**
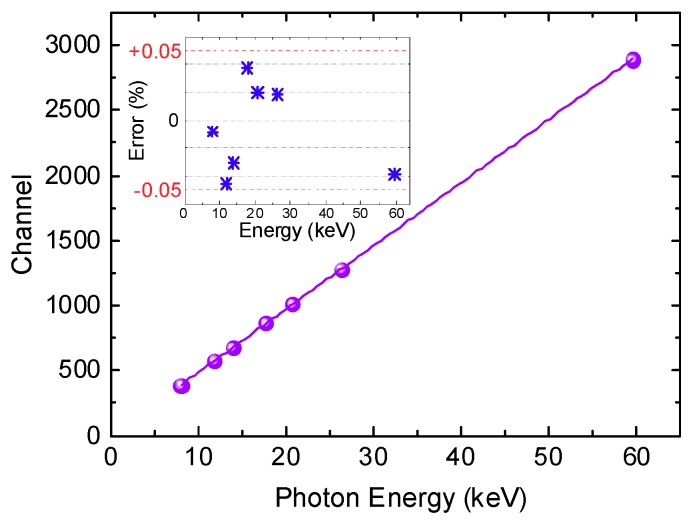
Analysis of linearity calculated on seven well-resolved peak lines, as shown in Figure 9. The percentage error from linearity is below ±0.05%.

The analysis of linearity calculated on seven well-resolved peak lines at 8.0, 11.87, 13.94, 17.8, 20.8, 26.35 and 59.54 keV is shown in Figure 10. The percentage error from linearity is below ±0.05%.

#### 3.2.2. Dependence of X-Ray Response on Detector Bias

**Figure 11:**
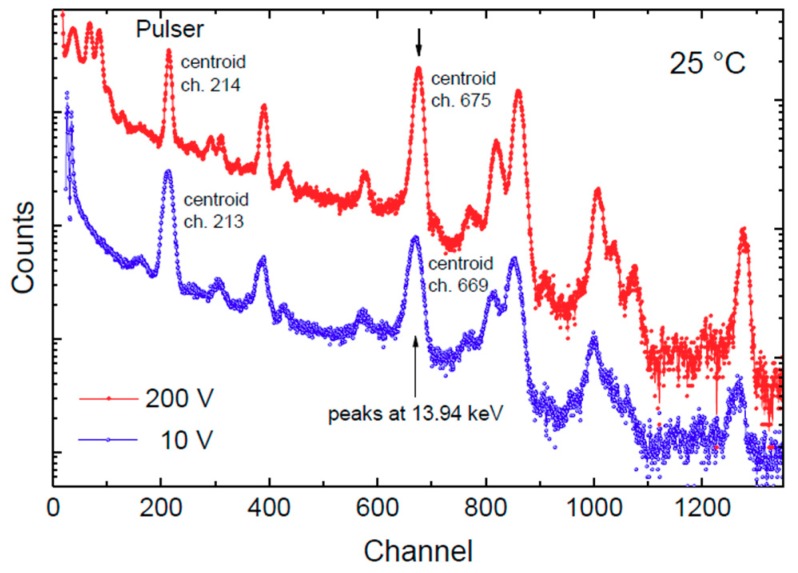
X-ray spectra from a ^241^Am source acquired at 25 °C and at 10 V (bottom) and 200 V (top).

**Figure 12:**
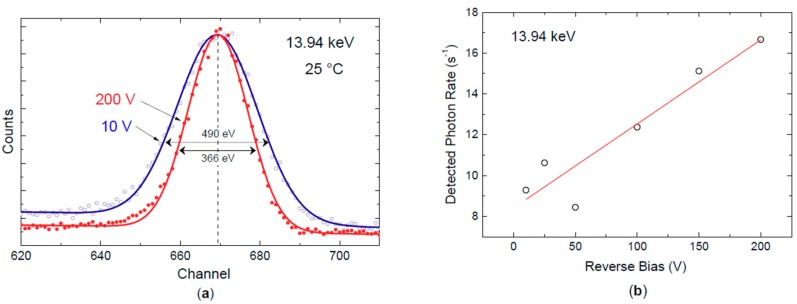
(**a**) Comparison between the two peaks at 13.94 eV obtained at 10 V and 200 V of applied voltage. The Gaussian symmetry without tails can be noticed; (**b**) Detected 13.94 keV photon rate as a function of the applied reverse bias.

The dependence of X-ray response on detector voltage was explored by biasing the detector from 10 V to 200 V at 25 °C and using a peaking time of 12.8 μs. A comparison between two spectra acquired at 10 V (bottom, blue) and 200 V (top, red) is shown in Figure 11. The pulser centroid is stable at both 10 V and 200 V. The peak at 13.94 keV shows a small shift of six channels (from 669 at 10 V to 675 at 200 V) which corresponds to 124 eV (Figure 12). The two peaks, as compared in Figure 12a, show a Gaussian symmetry without any tails. This experiment shows that SiC detectors can be operated in a wide range of bias voltages without suffering from a strong performance loss. Figure 12b shows the detection rate of the 13.94 keV photon peak at six different applied reverse-bias voltages, *V_b_*. As expected, the photon rate increases with the square root of *V_b_* due to the widening of the active region (depletion layer).

#### 3.2.3. High Statistics and Temperature Dependence

**Figure 13:**
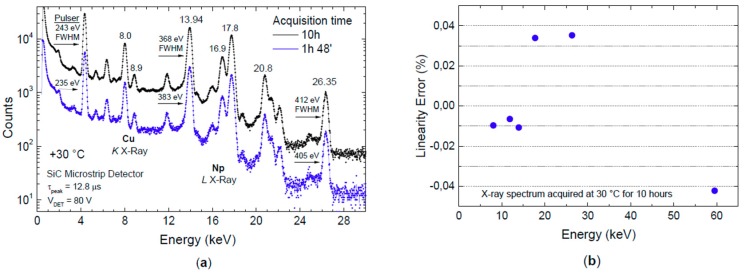
(**a**) X-ray spectra from ^241^Am source after almost 2 and 10 h of acquisition at 30 °C in a thermostatic chamber. The very small broadening of pulser and emission lines demonstrates very good stability of the detector response to X-ray exposure; (**b**) linearity error after 10 h of acquisition based on *K* Cu and *L* Np X-ray monoenergetic lines.

Figure 13 shows the results obtained by acquiring an X-γ-ray spectrum for 10 h at 80 V reverse-bias condition, and maintaining the thermostatic chamber at a constant temperature of 30 °C. Figure 13a compares spectra acquired after almost 2 h and after 10 h. The very small broadening of the pulser and emission lines should be noted, which demonstrates very good stability of the detector response to X-ray exposure. The analysis of linearity on *K* Cu and *L* Np X-ray monoenergetic lines shows a very small linearity error within ±0.04% after 10 h of acquisition (Figure 13b).

**Figure 14:**
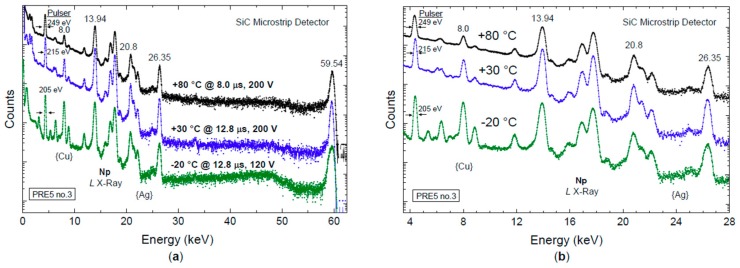
(**a**) Comparison between three X-ray spectra acquired at −20 °C, +30 °C, and +80 °C in the range 0–60 keV; (**b**) Detail of the X-ray spectra in the range 4–28 keV. Note a small broadening of the pulser line by increasing the operating temperature.

Figure 14 shows a comparison between X-ray spectra acquired at three different temperatures, i.e., −20 °C, +30 °C, and +80 °C. As expected, the width of pulser and emission lines increased by increasing the operating temperature: the FWHM of the pulser changed from 205 eV at −20 °C, to 215 eV at +30 °C, and 249 eV at +80 °C. It is worth noticing the small broadening of the lines at +80 °C, which demonstrates the suitability of our microstrip detector to be used at high temperatures with very good stability of the detector response.

## 4. Discussion

Two SiC-based microstrip detectors were fully characterized in a wide temperature range by means of electrical and spectroscopic measurements. A high stability of the detector response as a function of operating temperature as well as of applied voltage was widely demonstrated. 

The very low leakage currents (current densities) from about 2 fA (4 pA/cm^2^) at 25 °C to 620 fA (1.2 nA/cm^2^) at 107 °C are among the best values measured on SiC detectors, and more than one order of magnitude lower than most silicon detectors [30,31]. Since the shot noise of the leakage current is a significant noise contribution in a radiation spectroscopy system, we can say that our SiC detectors allow the achievement of high signal-to-noise ratios. A good isolation between adjacent strips was demonstrated by the high value of the measured interstrip resistance of 5.3 TΩ, confirming that possible latent currents from adjacent strips can be considered negligible.

A very good doping uniformity of the whole epitaxial layer was also demonstrated. For the first time, a full depletion of 124 µm was reached, polarizing the detector at 600 V, and a mean value of <N_D_> = 5.2 × 10^13^ cm^−3^ was determined. In comparison with previous studies, this result is the best observed [32].

The X-ray spectra acquired from a ^241^Am source at different voltages, temperatures, and exposure times showed high stability and a high spectroscopic resolution under all tested experimental conditions. 

Different voltages were used to verify the effect of the applied bias voltage on the device performance. The spectroscopic response of our SiC detector does not significantly depend on the bias voltage, as shown in Figure 11, where two extreme bias voltages (10 V and 200 V) were used. No tails in the spectral lines were observed, which means that no significant charge trapping occurred in these devices. Remarkably, no strong performance loss was observed at 10 V, and substantially no difference was observed under operation between 80 V and 200 V. The possibility of using a lower voltage without losing significant information is an advantage for those applications wherein lower power consumption is desirable. The better resolution obtained at 200 V is due to a lower capacitance. We avoided operating the SiC detector above 200 V to prevent the risk of possible damages due to accidental breakdown or electrostatic discharge. Also, the exposure time to the ^241^Am source does not affect the spectroscopic performance of our SiC detector, as demonstrated by negligible differences in the peak resolution after almost 2 and 10 h of acquisition (Figure 13). This means that it is not necessary to wait for a long time before getting all the main information from the device.

Finally, it is worth noticing the high resolution and very good stability in the performance of our SiC microstrip detector between −20 °C and +80 °C (Figure 14), which pave the way for use in a wide range of applications that are prohibitive for other conventional semiconductor detectors.

## 5. Conclusions

Even considering an initial higher cost for the SiC material in comparison to Si or Ge, SiC-based semiconductor radiation detectors are advantageous for use in operating conditions under which conventional semiconductor detectors in Si or Ge cannot adequately perform. One overall advantage is the elimination of cryogenic or Peltier cooling systems that allows the fabrication of far more compact, more stable, lighter, and lower power radiation detector systems. This also implies, as a direct consequence, economic advantages that go beyond the mere costs for the material itself.

Our findings confirm the high quality and the good uniformity of the epitaxial layer used for manufacturing our SiC-based prototypes, as well as the suitability of such devices to be used as high resolution X-ray detectors over a wide range of operating temperatures.
